# Fano Resonance in CO_2_ Reduction Catalyst Functionalized Quantum Dots

**DOI:** 10.1021/jacs.4c14499

**Published:** 2025-03-21

**Authors:** Sara T. Gebre, Luis Martinez-Gomez, Christopher R. Miller, Clifford P. Kubiak, Raphael F. Ribeiro, Tianquan Lian

**Affiliations:** † Department of Chemistry, Emory University, Atlanta, Georgia 30322, United States; ‡ Department of Chemistry and Biochemistry, University of California, San Diego, 9500 Gilman Drive, MC 0358, La Jolla, California 92093, United States

## Abstract

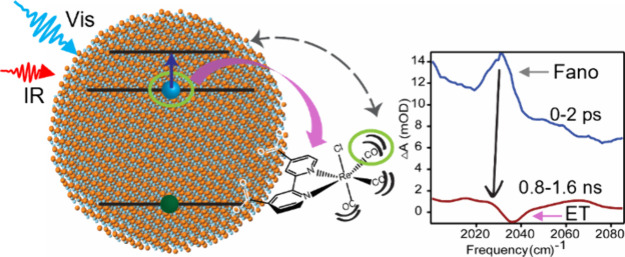

Molecular catalyst functionalized semiconductor quantum dots (QDs) are a promising modular platform for developing novel hybrid photocatalysts. The interaction between adsorbed catalyst vibrations and the QD electron intraband absorption can influence the photophysical properties of both the QD and the catalysts and potentially their photocatalysis. In CdSe QDs functionalized by the CO_2_ reduction catalyst, Re(CO)_3_(4,4’-bipyridine-COOH)Cl, we observe that the transient Fano resonance signal resulting from coupling of the catalyst CO stretching mode and the QD conduction band electron mid-IR intraband absorption appears on an ultrafast time scale and decays with the electron population, irrespective of the occurrence of photoreduced catalysts. The Fano asymmetry factor increases with an increase in the adsorbed catalyst number and a decrease in QD sizes. The latter can be attributed to an enhanced charge transfer interaction between the more strongly quantum-confined QD conduction band and catalyst LUMO levels. These results provide a more in-depth understanding of interactions in excited QD-catalyst hybrid photocatalysts.

## Introduction

Semiconductor (SC) nanomaterials have emerged as a new class of materials with many potential applications in photocatalysis,^1−5^ photodetectors,^6−8^ and solar cells.^9−12^ The optical properties of SC quantum dots (QDs) can be manipulated by altering their size and composition.^13−19^ This allows for the design of nanomaterials with specific energy levels and differing surfaces which affects their charge carrier lifetimes, making them suitable for the aforementioned applications. The integration of SC QDs with molecular catalysts facilitates the prospect of enhancing processes such as photodriven CO_2_ reduction and H_2_ generation, and nanoparticle-molecular catalyst complexes have emerged as promising hybrid photocatalysts, circumventing the disadvantages of molecular catalysts on their own.

Several catalysts exhibit distinctive vibrational signatures in the infrared region that allow for monitoring key intermediates involved in their catalytic reactions.^20^ Additionally, semiconductor nanocrystals (NCs), specifically QDs, are known to have broad absorptions in the infrared, corresponding to intraband transitions in the conduction band. Oftentimes, ligands residing on the surface of QDs also have manifest vibrational signatures in the IR region.^21−24^ Thus, adsorbate vibrations and QD intraband excitations may overlap and interact, leading to the formation of a Fano resonance (FR).^25^ FR arises from the mixing of a discrete quantum state to a continuum; here these would correspond to a sharp vibrational mode from a catalyst or ligands and the broad absorption to the intraband transition of QDs, respectively. This phenomenon has been observed in various types of systems, most commonly in plasmonic nanomaterials.^26−29^ For example, Agrawal et al. investigated F and Sn codoped InO_3_ NCs with oleate ligands bound to the surface.^30^ They observed that the C–H bonds within the oleate can couple to the localized surface plasmon resonance of the NCs, resulting in a Fano line shape. FR usually manifests as a derivative shaped feature in IR spectra, but can vary depending on the Fano asymmetry parameter *q* which relates to the transition probabilities into the continuum and the hybridized discrete level and determines the shape of the observed feature.^25,31^ This type of coupling can also occur in QDs.

Recently, we have demonstrated that FRs emerge upon binding a Fe–Fe molecular catalyst ([Fe_2_(cbdt)(CO)_6_] to CdS nanorods (NRs) and CdSe QDs.^32^ Fano resonance coupling occurred between the three vibrational modes of the catalyst and the broad absorption from the NCs regardless of whether electron transfer was possible. Several studies have also demonstrated in literature that QD excitons can couple to the vibrational modes of their capping ligands, usually long chain organic molecules such as oleic acid, in the near IR region.^21,22^ Upon exchanging the native ligands, it has been shown that charge carrier dynamics can be affected, particularly intraband relaxation of hot carriers.^18,19,33−35^ This presents the intriguing prospect of vibrational coupling, in this specific case, FR coupling, being able to affect charge transfer processes within NC-molecular catalyst complexes.

**Scheme 1:**
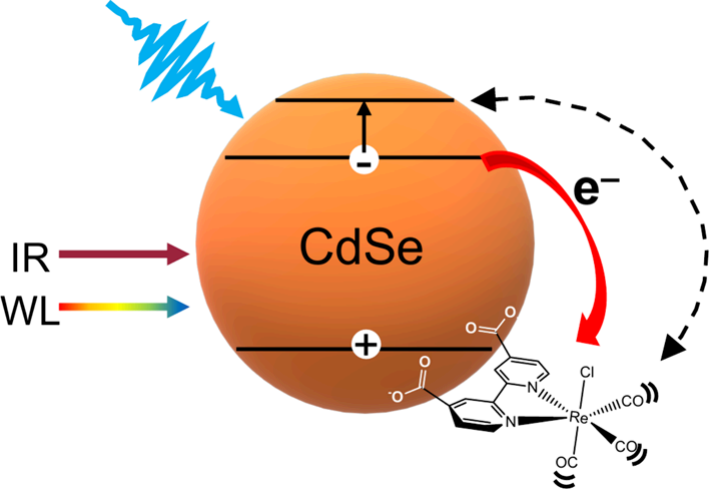
Illustration Demonstrating Exciton Formation after Visible Pump Excitation After the exciton is formed the 1S electron can be transferred to the ReC0A complex. The 1S electron can also be promoted to the 1P state by the IR probe, in which case, would result in Fano resonance coupling with the catalyst CO stretching modes.

Despite the reports of Fano resonance between QDs and adsorbates, the mechanism for the Fano coupling remains unclear. Herein, we use a well-known CO_2_ reduction catalyst, Re(CO)_3_(4,4’-bipyridine-COOH)Cl (ReC0A) at several concentrations, and bound to QDs of varying sizes, to investigate the effect of catalyst loading and QD size on the observed transient FRs (Scheme 1). We use both transient visible (TA) and infrared (TRIR) absorption spectroscopies to monitor electron transfer between the QD and complex, as well as the shape of the FR under these different conditions. We find that the Fano asymmetry parameter *q* increases with catalyst concentration but decreases with increasing QD size. These observations are rationalized using a recently developed vibronic Fano model whereby charge-transfer interactions between the QD and the catalyst mediate an effective interaction between the QD intraband transition and the molecular vibrational polarization. This effective interaction creates a vibrational energy relaxation pathway through coupling to QD electronic transitions. Understanding these interactions is crucial to designing hybrid photocatalysts similar to our QD-ReC0A system. Because both molecular vibrations, strong broad intraband absorptions in semiconductors, and charge transfer interaction are present in all semiconductor/molecular catalyst hybrid materials, this charge-transfer interaction induced Fano resonance is likely a general phenomenon. Finally, the Fano resonance signatures offer a new method to probe weak vibrations significantly enhanced through coupling to electronic transitions.^36,37^ Overall, the results provide new insights into NC-catalyst interactions and their signatures in ultrafast pump–probe spectra.

## Results and Discussion

### Characterization of Quantum Dots

**Figure 1:**
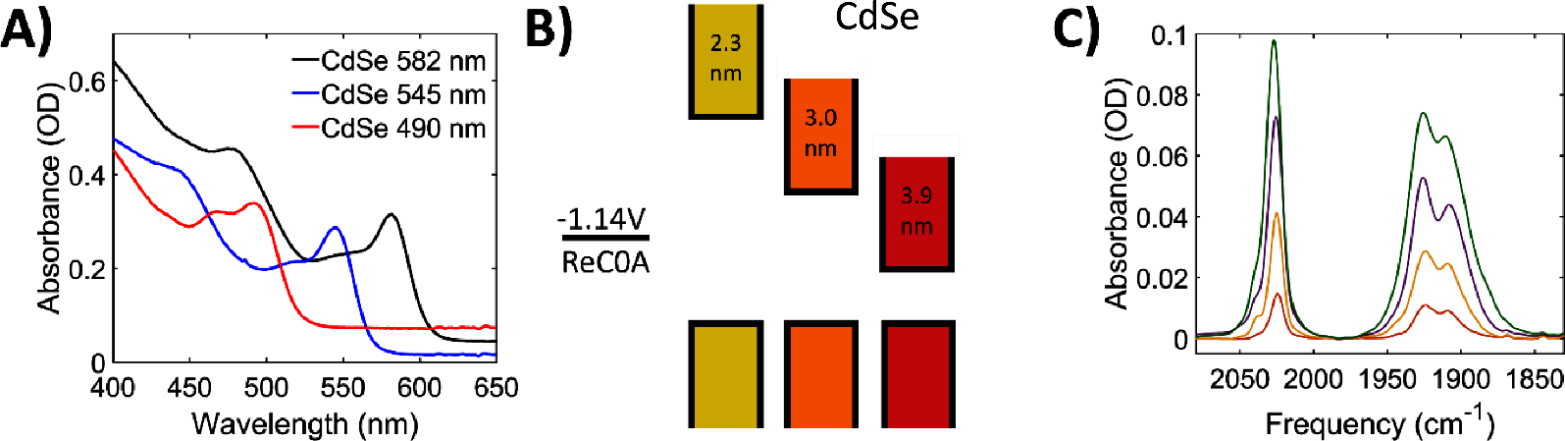
(A) UV–vis spectra of the three CdSe QDs demonstrating their exciton band positions. Corresponding sizes are 2.3 (CdSe490), 3.0 (CdSe545), and 3.9 (CdSe582) nm. (B) Energy band diagrams of each QD compared to the reduction potential of ReC0A. Lower bands correspond to the valence band for each nanoparticle, while the higher bands are the conduction bands. As the QD diameter increases, the band gap decreases. (C) Solvent subtracted FTIR spectra of CdSe490 with varying amounts of ReC0A bound to the surface (red: 0.25x, yellow: 0.5x, purple: 1x, green: 2x).

CdSe QDs were used in our present study because of their wide size tunability within the visible range. Several sizes of CdSe QDs were synthesized following previous literature,^38^ resulting in exciton band absorptions corresponding to sizes ranging from 2 to 5.6 nm in diameter. CdSe QDs were dispersed in heptane or hexane solutions, and UV–vis spectra and band diagrams for three QDs are shown in Figure 1a,b. Their exciton band absorptions are centered at 490, 545, and 582 nm, corresponding to 2.3, 3.0, and 3.9 nm diameters, which we refer to as CdSe490, CdSe545, and CdSe582, respectively.

Different concentrations of ReC0A were introduced to each size of CdSe QDs dispersed in hexane, as depicted in FTIR (Figure 1c). The molecular catalyst is not soluble in hexane, so the intensity of the vibrational mode absorption indicates the amount of ReC0A molecules bound to the QD surface, likely through their carboxylic acid ligands (Figure S1a). Average amounts of catalyst bound for each concentration are detailed in Table S1. The catalyst in acetonitrile (MeCN) has a visible absorption centered at ∼400 nm corresponding to the metal to ligand charge transfer (MLCT) state (Figure S1c).^39,40^ FTIR spectra of ReC0A in MeCN show three vibrational modes centered at 2025, 1921, and 1905 cm^–1^, corresponding to the symmetric, asymmetric, and out of phase symmetric CO stretches, respectively (Figure S1b). Upon addition to the QDs, the lower frequency modes slightly shift to 1925, and 1909 cm^–1^ (Figure 1c). Figure S2a,b shows FTIR of CdSe545 and CdSe582 with varying concentrations of ReC0A and demonstrate the same trend; as the added ReC0A concentration increases, more ReC0A binds to the QD. In the FTIR spectra shown in Figure 1c, the solvent contribution has been subtracted, as the solvent (hexane) also has multiple absorption bands in this spectral region, which can be seen in the unsubtracted FTIR shown in Figure S2c. The catalyst discrete vibrational modes will couple to the 1S to 1P intraband transition of the QD to generate FR signals.^41^ We focus on the high frequency mode centered at 2025 cm^–1^ in the transient IR experiments as it is easier to distinguish it from the solvent modes.

### Electron Transfer Rates after Visible Excitation

**Figure 2:**
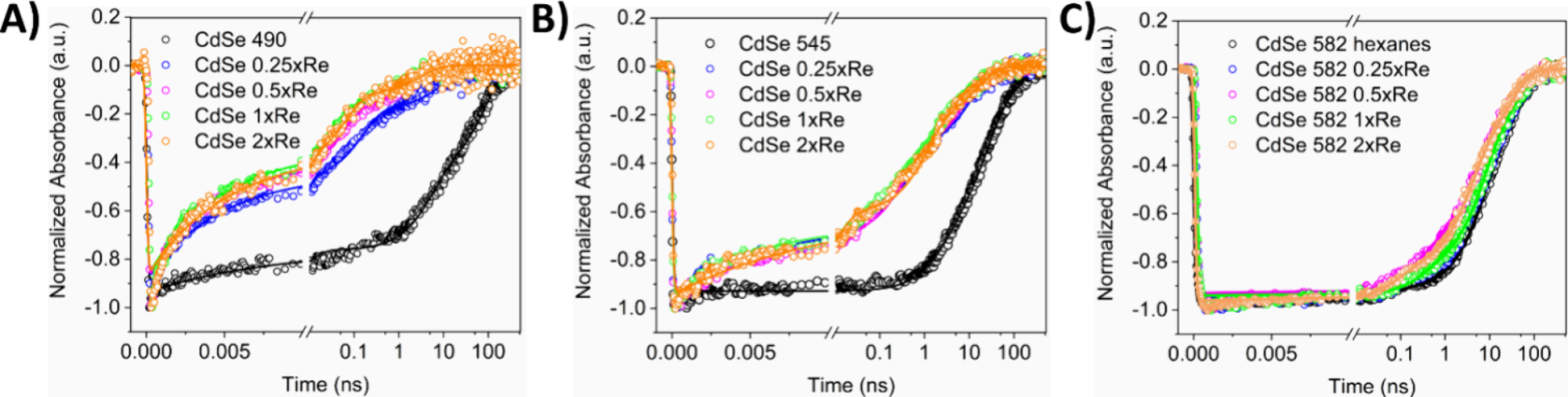
Fitted kinetics of CdSe QDs with varying amounts of ReC0A bound, probed at their corresponding ground state bleach frequency. (A) CdSe490. The addition of ReC0A to the QD results in faster bleach recovery indicating electron transfer. (B) CdSe545. At this QD size, the CB edge is still more negative than the ReC0A reduction potential. Slower bleach recovery compared to CdSe490 indicates electron transfer does not occur as quickly. (C) CdSe582. Concentration-independent kinetics implies negligible electron transfer.

Transient absorption (TA) experiments were performed to elucidate the electron transfer properties of CdSe QDs with varying concentrations of ReC0A and were conducted under 475 nm excitation, to avoid direct excitation of the catalyst. The first panel in Figures S3–S5 shows TA spectra for each individual QD in heptane, CdSe490, CdSe545, and CdSe582, respectively. After excitation, ground state bleaching appears in each sample due to the filling of the 1S conduction band (CB) state.^42^ Triexponential fits resulted in amplitude weighted average time constants that were on the order of nanoseconds shown in Table S2. Figure 2 shows the kinetic traces for each sample probed at the center of the QD bleach signals. For CdSe490, the exciton bleach (XB) recovery occurs significantly faster upon addition of 0.25xRe (0.69 ns), indicating electron transfer to the catalyst (Figures 2 and S3). As more ReC0A is added, the bleach recovery lifetime is slightly faster in comparison to the 0.25xRe case. The same trend is observed for CdSe545; however, compared to CdSe490 with the same number of molecules, the lifetimes of exciton bleach recovery in CdSe545 are longer (between 0.99 and 1.44 ns), indicating slower electron transfer to the catalyst. This is due to the increased size of the QD compared to CdSe490. As the QD size increases, the band gap decreases. This lowers the position of the CB band edge corresponding to a decrease in the driving force for electron transfer to the catalyst (Figure 1b). For CdSe582, addition of ReC0A shows only minor decrease of the exciton bleach recovery time (Figure 1c and Table S2), indicating much slower electron transfer, which is attributed to further decrease of the driving force of electron transfer from these larger QDs. Additionally, there exists electronic coupling between the QD and catalyst. However, the driving force effect dominates with regards to the electron transfer rates. Electron transfer leads to a singly reduced catalyst known to have a weak absorption at approximately 520 nm.^43^ This is not observed, due to the large bleach signal of the QDs which most likely obscures the much smaller signal of the reduced catalyst.

### Fano Resonance as a Function of Catalyst Concentration

**Figure 3:**
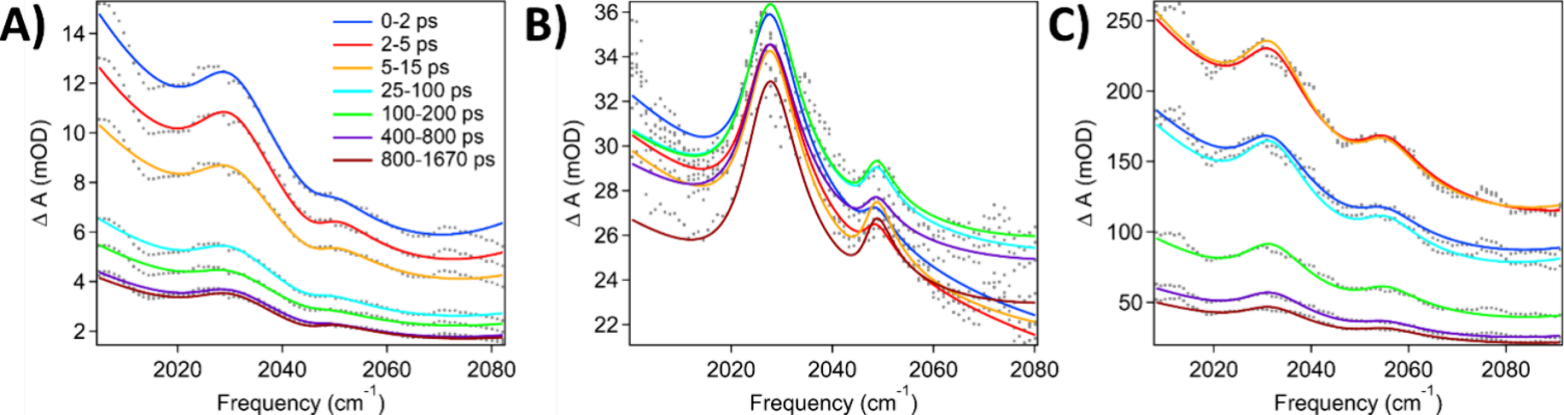
TRIR spectra of CdSe QDs in hexane. (A) CdSe490 (B) CdSe545 (C) CdSe582. Absorption peaks at ∼2030 and 2050 cm^–1^ suggest solvent vibrations couple with the QD intraband transition.

Time resolved infrared spectroscopy (TRIR) was used to determine the interaction between the QDs and varying concentrations of catalyst in hexane. We first measured the transient IR spectra of QDs in hexane without the presence of ReC0A catalyst. As shown in Figure 3a–c for CdSe490, CdSe545, and CdSe582, respectively, upon 475 nm excitation, there is an instantaneous formation of a broad IR absorption in the probed region of 2000–2080 cm^–1^ region, which has been attributed to the 1S to 1P intraband transition of electrons in the CdSe QD CB.^32^ Furthermore, we observed two large positive absorption features at 2030 and 2050 cm^–1^ that correspond to solvent (hexane) absorption bands (Figure S2c). The amplitudes of these vibrational features are proportional to that of the broad 1S-1P intraband absorption, suggesting that it is unlikely to be caused by shift of solvent bands caused by heating or refractive index changes. We attribute these features to Fano resonance between the narrow solvent vibrational modes and the broad photoinduced IR 1S to 1P transition in the CdSe QD CB.^32^ This coupling most likely proceeds via a direct dipole–dipole interaction. A simple estimate of the Fano asymmetry factor assuming the QD intraband transition dipole moment is 3 orders of magnitude greater than the solvent vibrational transition and a distance in the range 1–10 nm gives a solvent-QD dipole–dipole Fano asymmetry factor *q* of order 1–10. Note that such solvent-adsorbate FR has previously been observed by Herliyh et al. with a discrete surface Ti–O vibration interacting with broad water librational modes.^37^ Upon the introduction of ReC0A to the QD solutions, the TA spectra show much larger positive bands at 2025 cm^–1^ in addition to similar positive solvent Fano resonance features (Figure S6). The substantial increase in the amplitude of Δ*A* at 2030 cm^–1^ (Figures S8 and S9) relative to the pure QD-hexane solutions provide evidence for significant catalyst-QD interaction. Although the FR originating from the coupling between ReC0A and the QDs is on top of the solvent absorption, the second peak at ∼2050 cm^–1^ corresponds mainly to a solvent mode.

The Fano parameter, *q* provides a measure of the asymmetry in the FR line shape. Microscopically, *q* is directly proportional to the transition probability into hybrid (molecular-QD) discrete states embedded in the intraband QD continuum, and inversely proportional to the transition probability into the unperturbed continuum states resonant with the catalyst vibrational mode.^31^ To determine the value of *q*, all TRIR spectra were globally fitted with the following equation (eq 1)^25,32,37^:
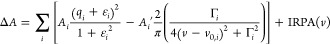
1where the first and second terms correspond to the absorption due to the formation of the Fano resonance and the (ground-state bleach) Lorentzian absorption, respectively. The IRPA term corresponds to the IR signal originating from the QD intraband electronic transition. *A*_*i*_ and  are the amplitudes of the Fano and Lorentzian terms, respectively. *q*_*i*_ is the Fano asymmetry parameter, Γ_*i*_ is the width of the IR transition, and *v*_0,*i*_ is the peak position of the catalyst vibrational feature. ε_*i*_ is the detuning term ε_*i*_ = 2(*v* – *v*_0,*i*_)/Γ_*i*_.

Since hexane appears to also couple to the QDs, we first globally fit the data to eq 1 with two Fano and two Lorentzian terms corresponding to each peak, 2030 and 2050 cm^–1^, to also obtain *q* values for the coupling of both solvent vibrations with the QD. For QD-ReC0A complexes, because the solvent and ReC0A CO symmetric mode absorption overlap, we subtracted the solvent peaks from each QD-ReC0A sample spectrum by using the solvent-QD *q* values and other parameters from the solvent fits. This is described in detail in the Supporting Information (Section S4). Hereafter, all spectra shown have been solvent subtracted. It should be noted that hexane is the optimal solvent for this study among many that we have tried because of the following requirements: (1) the QD is soluble but ReC0A is not soluble in the solvent, so that all dissolved ReC0A molecules are on the QD, and (2) the solvent should have minimal IR absorption in the CO stretching mode region.

**Figure 4:**
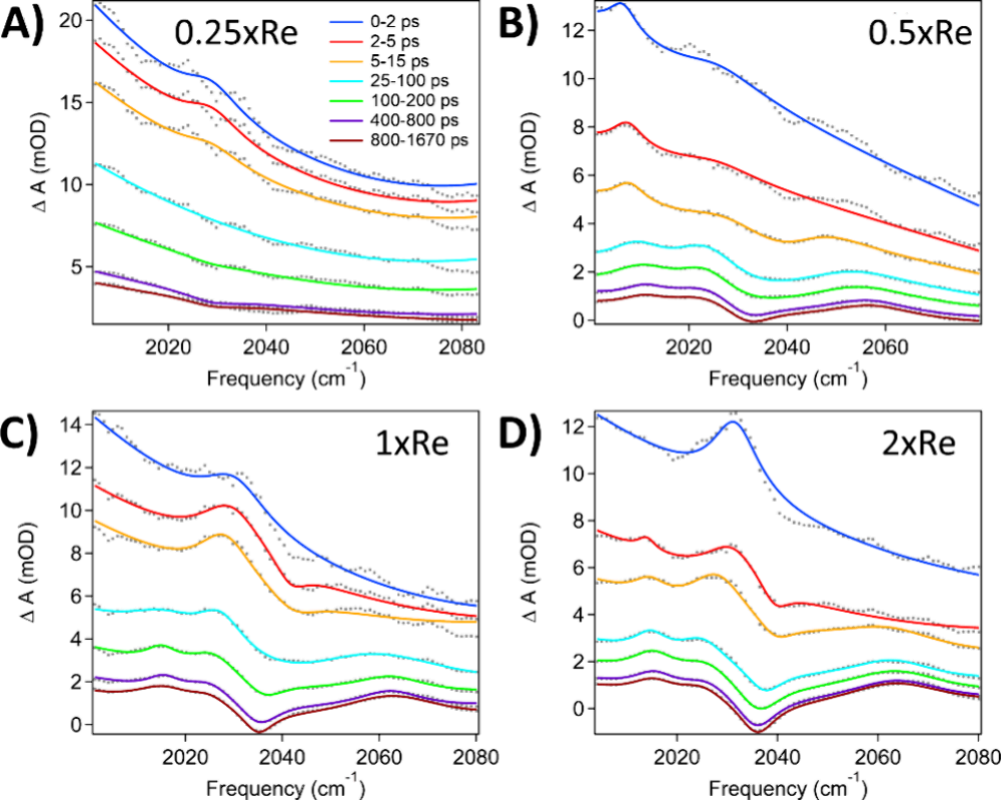
Transient IR spectra of CdSe490 with varying amounts of ReC0A. Since the singly reduced ReC0A species begins to appear as the FR signal decays, two more Lorentzians were used to fit the peak at ∼2013 cm^–1^ and bleach at 2035 cm^–1^. (A) CdSe490-0.25xRe. (B) CdSe490-0.5xRe. (C) CdSe490-1xRe. (D) CdSe490-2xRe.

For each QD, as the amount of bound ReC0A increases, the amplitude of the FR signal increases, suggesting enhanced oscillator strength for the transition into the catalyst discrete mode. Figure 4 shows CdSe490 without ReC0A, and with increasing amounts of ReC0A, demonstrating this trend. We attribute this to the total strength of the CO vibrational oscillator on the QD surface. With more catalyst bound to the surface, there are more CO modes, and therefore a larger dipolar polarization density. This increase in the oscillator strength of the catalyst modes on the QD surface leads to an expected increase in *q* with catalyst concentration. As the QD excited-state decays via charge transfer or nonradiative decay, the FR amplitude simultaneously decreases, indicating the essential role of the conduction band electron in promoting the effective coupling to the catalysts. After the FR signal decays, a peak at ∼2013 cm^–1^ and a negative feature at 2035 cm^–1^ suggest that ReC0A has been singly reduced.^41,44,45^ This confirms that the FR signal obstructs the singly reduced signal. Between the 1x and 2xRe concentrations, the amplitudes of the signals generated by the reduced species get larger, indicating more ReC0A is reduced as we have observed with Cd_3_P_2_–ReC0A in a previous paper.^44^ However, the 0.5xRe sample seems to be the outlier. We believe the *q* value is lower compared to the 0.25xRe case because the solvent could not be completely subtracted from the raw data (the presence of a small feature around 2055 cm^–1^), whereas the subtraction for the other samples was able to completely eliminate the solvent contribution. The growth in the FR signal with higher ReC0A concentration was likewise noted in the case of the other two studied QDs, CdSe545 (Figure S8) and CdSe582 (Figure S9). Nevertheless, due to the slower electron transfer kinetics compared to the smallest QD, the CO stretching modes of the singly reduced species were not observed. The fits for each sample spectra agree well with the data. Table 1 shows the *q* values obtained for each sample and demonstrate *q* is increasing as a function of catalyst concentration for each QD size.

**Table 1:** Fano Asymmetry Parameters *q* for Each QD and ReC0A Concentration^a^

	0.25xRe	0.5xRe	1xRe	2xRe	4xRe
CdSe490	10.3 ± 1.9	5.24 ± 0.36	21.9 ± 4.2	41.4 ± 12.1	
CdSe545		15.5 ± 3.5	20.6 ± 3.17	32.7 ± 5.2	66.4 ± 15.3
CdSe582	10.6 ± 1.9	12.3 ± 1.4	20.2 ± 4.5	23.2 ± 4.4	

a With higher ReC0A concentrations, *q* is observed to increase.

### Fano Resonance as a Function of QD Size

**Figure 5:**
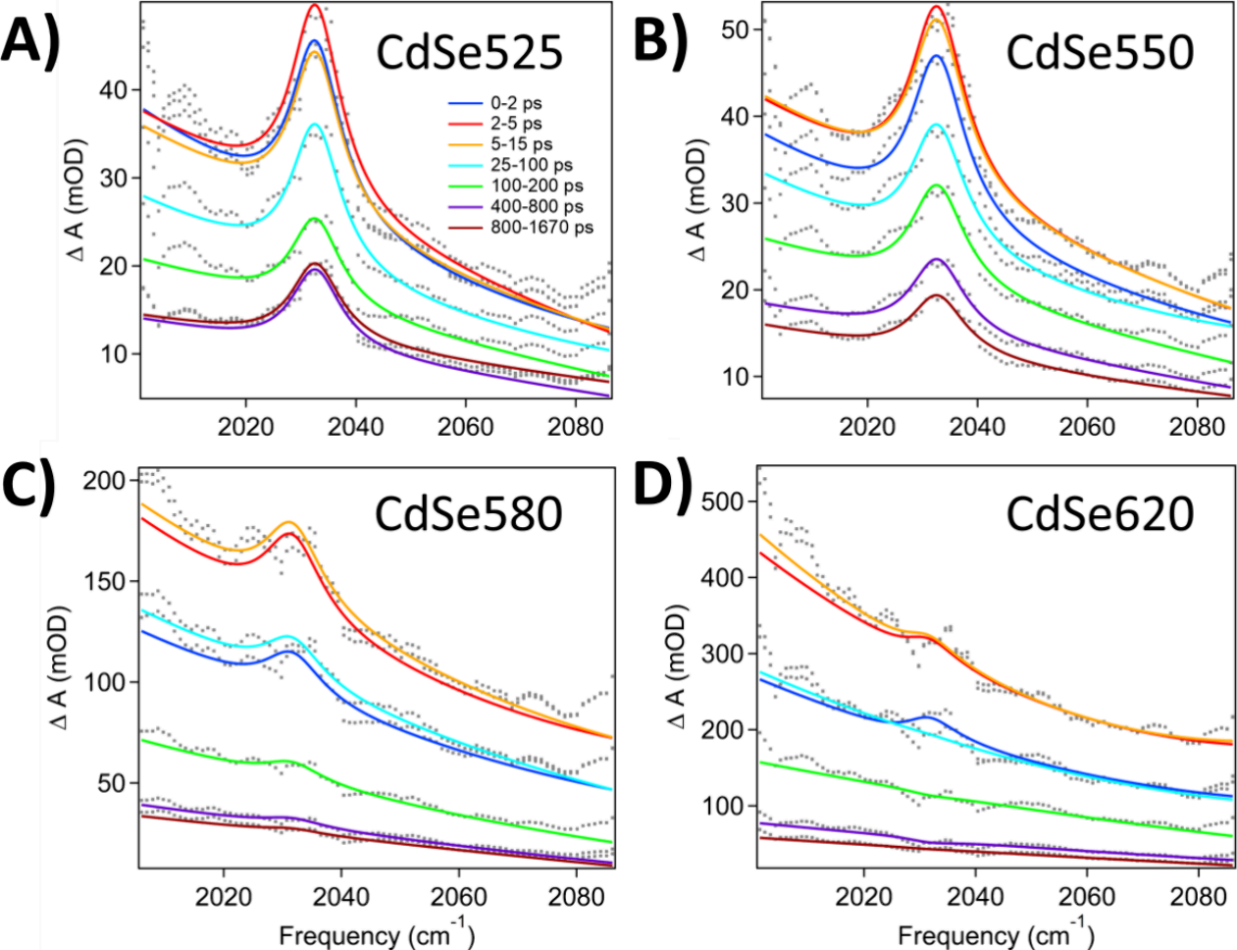
TRIR spectra of CdSe QD-ReC0A complexes of varying QD sizes after solvent subtraction. (A) CdSe525-2xRe. (B) CdSe550-2xRe. (C) CdSe580-2xRe. (D) CdSe620-2xRe.The same concentration of ReC0A (2x) was added to each QD, resulting in a similar number of adsorbates (Table S3) to minimize the catalyst concentration dependence effect. The above spectra show the relative amplitude of FR vs QD 1S-1P absorption increases at smaller QD size, reflecting the size dependent FR coupling in these samples.

We also investigated the effect of QD size on the FR originating from its coupling to the molecular catalyst. It is not explicitly apparent from the spectra for CdSe490 and CdSe582 at a concentration of 2xRe, that there is a significant difference in the FR amplitude. However, our analysis shows Fano asymmetry factors that reveal considerable size dependence. Additionally, the number of adsorbates per QD for both of these concentrations varies greatly (Table S1, CdSe490-2xRe: 8.4 molecules vs CdSe582-2xRe: 25.6 molecules). To minimize the contribution from the concentration dependent effect on the FR and more clearly investigate any QD size dependence, four new QDs, CdSe525 (2.6 nm), CdSe550 (3.0 nm), CdSe580 (3.8 nm), and CdSe620 (5.6 nm), were synthesized. The highest concentration of ReC0A (2x) was added to each, with their FTIR spectrum shown in Figure S11. Table S3 shows that the number of adsorbates per QD are more similar compared to the values in Table S1 for the same ReC0A concentration (2xRe). Upon 475 nm excitation of the QDs in pure hexane, as seen previously, we observe two positive features at approximately 2030 and 2050 cm^–1^, corresponding to the solvent absorption (Figure S10). Upon addition of ReC0A, the solvent response was still present, and the intensity of the 2030 cm^–1^ peak increased, as previously observed. The solvent FR was fitted to eq 1 and the values obtained were used to subtract the solvent contribution from the QD-ReC0A samples as described previously. The subtracted QD-ReC0A FRs were then globally fitted to the same equation to obtain *q* values (Table 2). Comparing both the FR amplitudes of each sample in Figures 5 and S12, as well as the corresponding asymmetry factors, there appears to be a basic trend. As the size of the QD increases, both the FR amplitude, and the asymmetry factor *q* decrease. Since FR strength scales with the number of bound molecules, we can normalize the FR *q* value by dividing by the calculated number of adsorbates on the surface to get the coupling per adsorbate, which demonstrates the same trend.

**Table 2:** Fano Parameter (*q*) Values for Each QD-2xRe Complex

	CdSe525	CdSe550	CdSe580	CdSe620
2xRe	29.8 ± 7.3	22.9 ± 3.6	20.2 ± 6.8	4.41 ± 1.1
normalized FR	1.37	1.32	0.79	0.159

We attribute the decrease of *q* with QD size primarily to the reduced extent of the QD electronic wave function surface penetration. Since QDs are quantum confined, in comparison to bulk semiconductors, they have larger electronic wave function amplitudes at longer distances from the surface.^46,47^ It is well-known that as QD size increases, a simultaneous decrease occurs for the band gap and the electronic wave function amplitude away from the surface due to the quantum confinement effect.^13,14,48^ As shown in computations by Zhu et al., the radial distributions for electron and hole surface densities decrease with larger QD size.^15^ It follows that as the QD size increases, the electronic wave function decays faster away from the surface. This feature leads to a decrease in the electronic charge-transfer coupling between the QD and catalyst CO vibrational modes promoted via its vibronic coupling with the catalyst LUMO (see below). A reduced spatial overlap between the molecular LUMO and the intraband electronic excited-state leads to a reduced Fano hybridization and a weaker FR signal with a smaller vibrational oscillator strength and asymmetry parameter *q,* as observed here.

The given qualitative analysis of the Fano asymmetry factor dependence on the QD size is supported by our microscopic model, which we describe mathematically and in detail in ref (49). and summarize here. First, recall that according to the FR theory,^31,49^*q* is proportional to the discrete oscillator absorption intensity and decreases with stronger interaction between the discrete and continuum modes. For the examined QD-catalyst system, the molecular vibration line width is primarily determined by intramolecular interactions and is only weakly affected by the interaction with the QD. Therefore, QD size variations primarily affect *q* through the effect of the excited QD on the catalyst’s vibrational oscillator strength.

**Figure 6:**
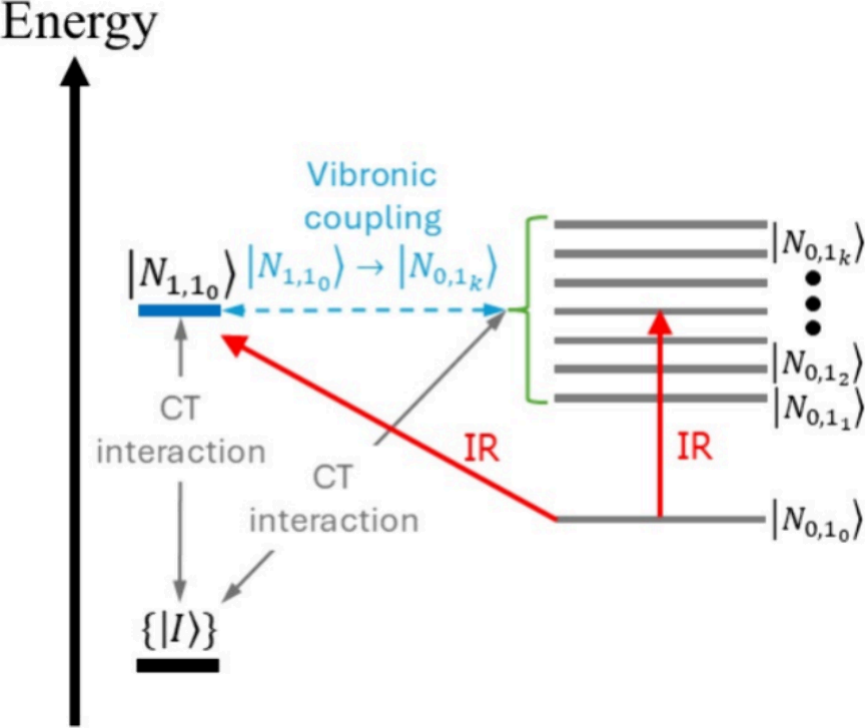
Schematic illustration of the proposed effective interaction between quantum dot (QD) intraband polarization and molecular vibrational resonance mediated by charge-transfer interactions. The UV pump generates the state |*N*_0,1_0__⟩ corresponding to the adsorbate-QD electronic state where both are neutral, the QD is in the 1S state and the relevant CO stretch is in the ground-state *v* = 0. The IR probe field induces electronic transitions from |*N*_0,1_0__⟩ into the QD 1P state (represented in the figure by the dense manifold of state {|*N*_0,1*_k_*_⟩, *k* ≠ 0}) and adsorbate vibrational excitation into its *v* = 1 state (represented by |*N*_1,1_0__⟩). In our model, the vibronic interaction between the QD 1P state and the CO stretch leading to the observed Fano resonances is mediated by ionic states {|*I*⟩} (the QD is positively charged and the adsorbate is negatively charged) coupled via charge-transfer (CT) interactions to the near-resonant states |*N*_0,1*_k_*_⟩ (QD 1P, CO *v* = 0) and |*N*_1,1_0__⟩ (QD 1S, CO *v* = 1) accessed via IR excitation. In summary, the effective coupling between the QD 1S → 1P and adsorbate *v* = 0 → 1 infrared polarizations emerges from the CT interactions between the molecular system and the excited QD.

In our proposed mechanism, charge-transfer interactions between the excited QD and the catalyst (i) mediate an effective vibronic coupling between the QD intraband polarization and the molecular vibrational resonance ultimately leading to the observed FRs and (ii) control the change in molecular vibrational oscillator strength induced by excitation of the QD (Figure 6). The second effect controls the observed behavior of *q* with system size.

Quantitatively, perturbation theory shows that QD-catalyst charge transfer interactions create ionic character in the molecular ground-state, modifying the vibrational oscillator strength quadratically with the charge transfer coupling strength. This interaction depends strongly on the QD conduction band-catalyst LUMO orbital overlap, which decreases with QD size, in direct agreement with the observed reduction in *q* for larger QDs. As in our previous discussion of the solvent-QD FRs, estimates of *q* obtained from dipole–dipole interactions are too low and unable to explain the reported size-dependence of the Fano asymmetry factor reported here.

## Conclusions

In conclusion, we have investigated how the Fano resonance between excited QDs and adsorbed molecular CO_2_ reduction catalysts depends on the number of adsorbed catalysts and the QD size. We have demonstrated that the FR signal increases with catalyst loading, which is induced by increased total oscillator strength of catalyst vibrations embedded in the QD intraband absorption continuum. In addition, we observed that increasing the QD diameter results in a decreasing FR signal, which is attributed to the reduced charge transfer interaction between larger QDs and catalysts because smaller quantum confinement in the QD decreases the degree of overlap between its 1S level and the ReC0A LUMO. These results have provided us with a deeper understanding of how FR is affected by various experimental conditions and lend us further insight into the interactions between QDs and their surface bound species.

## Supporting Information Available

Experimental details as well as sample characterization, steady-state UV–vis, FTIR, TA, and TRIR data from additional experiments, and details of the solvent subtraction process for the TRIR data (PDF)

## References

[ref1] Wang C., Chapter 9 - Quantum Dots for Visible-Light Photocatalytic CO_2_ Reduction. In Novel Materials for Carbon Dioxide Mitigation Technology; Shi F.; Morreale B., Eds.; Elsevier: Amsterdam, 2015; pp 269–295.

[ref2] Moore G. F.; Brudvig G. W. Energy Conversion in Photosynthesis: A Paradigm for Solar Fuel Production. Annual Review of Condensed Matter Physics 2011, 2 (1), 303–327. 10.1146/annurev-conmatphys-062910-140503.

[ref3] Kamat P. V. Quantum Dot Solar Cells. Semiconductor Nanocrystals as Light Harvesters. J. Phys. Chem. C 2008, 112 (48), 18737–18753. 10.1021/jp806791s.

[ref4] Wu K.; Lian T. Quantum confined colloidal nanorod heterostructures for solar-to-fuel conversion. Chem. Soc. Rev. 2016, 45 (14), 3781–3810. 10.1039/C5CS00472A. 27043714

[ref5] Li Q.; Zhao F.; Qu C.; Shang Q.; Xu Z.; Yu L.; McBride J. R.; Lian T. Two-Dimensional Morphology Enhances Light-Driven H2 Generation Efficiency in CdS Nanoplatelet-Pt Heterostructures. J. Am. Chem. Soc. 2018, 140 (37), 11726–11734. 10.1021/jacs.8b06100. 30145886

[ref6] Đorđević N.; Schwanninger R.; Yarema M.; Koepfli S.; Yarema O.; Salamin Y.; Lassaline N.; Cheng B.; Yazdani N.; Dorodnyy A.; Fedoryshyn Y. M.; Wood V.; Leuthold J. Metasurface Colloidal Quantum Dot Photodetectors. ACS Photonics 2022, 9 (2), 482–492. 10.1021/acsphotonics.1c01204.

[ref7] Livache C.; Martinez B.; Goubet N.; Gréboval C.; Qu J.; Chu A.; Royer S.; Ithurria S.; Silly M. G.; Dubertret B.; Lhuillier E. A colloidal quantum dot infrared photodetector and its use for intraband detection. Nat. Commun. 2019, 10 (1), 2125 10.1038/s41467-019-10170-8. 31073132 PMC6509134

[ref8] Guo R.; Zhang M.; Ding J.; Liu A.; Huang F.; Sheng M. Advances in colloidal quantum dot-based photodetectors. Journal of Materials Chemistry C 2022, 10 (19), 7404–7422. 10.1039/D2TC00219A.

[ref9] Kirmani A. R.; Luther J. M.; Abolhasani M.; Amassian A. Colloidal Quantum Dot Photovoltaics: Current Progress and Path to Gigawatt Scale Enabled by Smart Manufacturing. ACS Energy Letters 2020, 5 (9), 3069–3100. 10.1021/acsenergylett.0c01453.

[ref10] Kamat P. V. Quantum Dot Solar Cells. The Next Big Thing in Photovoltaics. J. Phys. Chem. Lett. 2013, 4 (6), 908–918. 10.1021/jz400052e. 26291355

[ref11] Hu L.; Zhao Q.; Huang S.; Zheng J.; Guan X.; Patterson R.; Kim J.; Shi L.; Lin C.-H.; Lei Q.; Chu D.; Tao W.; Cheong S.; Tilley R. D.; Ho-Baillie A. W. Y.; Luther J. M.; Yuan J.; Wu T. Flexible and efficient perovskite quantum dot solar cells via hybrid interfacial architecture. Nat. Commun. 2021, 12 (1), 466 10.1038/s41467-020-20749-1. 33473106 PMC7817685

[ref12] Kim M. R.; Ma D. Quantum-Dot-Based Solar Cells: Recent Advances, Strategies, and Challenges. J. Phys. Chem. Lett. 2015, 6 (1), 85–99. 10.1021/jz502227h. 26263096

[ref13] Smith A. M.; Nie S. Semiconductor Nanocrystals: Structure, Properties, and Band Gap Engineering. Acc. Chem. Res. 2010, 43 (2), 190–200. 10.1021/ar9001069. 19827808 PMC2858563

[ref14] Alivisatos A. P. Semiconductor Clusters, Nanocrystals, and Quantum Dots. Science 1996, 271 (5251), 933–937. 10.1126/science.271.5251.933.

[ref15] Zhu H.; Lian T. Wavefunction engineering in quantum confined semiconductor nanoheterostructures for efficient charge separation and solar energy conversion. Energy Environ. Sci. 2012, 5 (11), 9406–9418. 10.1039/c2ee22679k.

[ref16] Kilina S.; Velizhanin K. A.; Ivanov S.; Prezhdo O. V.; Tretiak S. Surface Ligands Increase Photoexcitation Relaxation Rates in CdSe Quantum Dots. ACS Nano 2012, 6 (7), 6515–6524. 10.1021/nn302371q. 22742432

[ref17] Hines D. A.; Kamat P. V. Recent Advances in Quantum Dot Surface Chemistry. ACS Appl. Mater. Interfaces 2014, 6 (5), 3041–3057. 10.1021/am405196u. 24506801

[ref18] Peterson M. D.; Cass L. C.; Harris R. D.; Edme K.; Sung K.; Weiss E. A. The Role of Ligands in Determining the Exciton Relaxation Dynamics in Semiconductor Quantum Dots. Annu. Rev. Phys. Chem. 2014, 65 (1), 317–339. 10.1146/annurev-physchem-040513-103649. 24364916

[ref19] Schnitzenbaumer K. J.; Labrador T.; Dukovic G. Impact of Chalcogenide Ligands on Excited State Dynamics in CdSe Quantum Dots. J. Phys. Chem. C 2015, 119 (23), 13314–13324. 10.1021/acs.jpcc.5b02880.

[ref20] Turner J. J. Infrared vibrational band shapes in excited states. Coord. Chem. Rev. 2002, 230 (1), 213–224. 10.1016/S0010-8545(01)00471-4.

[ref21] Leger J. D.; Friedfeld M. R.; Beck R. A.; Gaynor J. D.; Petrone A.; Li X.; Cossairt B. M.; Khalil M. Carboxylate Anchors Act as Exciton Reporters in 1.3 nm Indium Phosphide Nanoclusters. J. Phys. Chem. Lett. 2019, 10 (8), 1833–1839. 10.1021/acs.jpclett.9b00602. 30925052

[ref22] Kennehan E. R.; Munson K. T.; Grieco C.; Doucette G. S.; Marshall A. R.; Beard M. C.; Asbury J. B. Exciton–Phonon Coupling and Carrier Relaxation in PbS Quantum Dots: The Case of Carboxylate Ligands. J. Phys. Chem. C 2021, 125 (41), 22622–22629. 10.1021/acs.jpcc.1c05803.

[ref23] Guyot-Sionnest P.; Wehrenberg B.; Yu D. Intraband relaxation in CdSe nanocrystals and the strong influence of the surface ligands. J. Chem. Phys. 2005, 123 (7), 074709 10.1063/1.2004818. 16229612

[ref24] Mack T. G.; Jethi L.; Andrews M.; Kambhampati P. Direct Observation of Vibronic Coupling between Excitonic States of CdSe Nanocrystals and Their Passivating Ligands. J. Phys. Chem. C 2019, 123 (8), 5084–5091. 10.1021/acs.jpcc.8b11098.

[ref25] Limonov M. F.; Rybin M. V.; Poddubny A. N.; Kivshar Y. S. Fano resonances in photonics. Nat. Photonics 2017, 11 (9), 543–554. 10.1038/nphoton.2017.142.

[ref26] Frontiera R. R.; Gruenke N. L.; Van Duyne R. P. Fano-Like Resonances Arising from Long-Lived Molecule-Plasmon Interactions in Colloidal Nanoantennas. Nano Lett. 2012, 12 (11), 5989–5994. 10.1021/nl303488m. 23094821

[ref27] Miroshnichenko A. E.; Flach S.; Kivshar Y. S. Fano resonances in nanoscale structures. Rev. Mod. Phys. 2010, 82 (3), 2257–2298. 10.1103/RevModPhys.82.2257.

[ref28] Luk’yanchuk B.; Zheludev N. I.; Maier S. A.; Halas N. J.; Nordlander P.; Giessen H.; Chong C. T. The Fano resonance in plasmonic nanostructures and metamaterials. Nat. Mater. 2010, 9 (9), 707–715. 10.1038/nmat2810. 20733610

[ref29] Wang M.; Krasnok A.; Zhang T.; Scarabelli L.; Liu H.; Wu Z.; Liz-Marzán L. M.; Terrones M.; Alù A.; Zheng Y. Tunable Fano Resonance and Plasmon–Exciton Coupling in Single Au Nanotriangles on Monolayer WS_2_ at Room Temperature. Adv. Mater. 2018, 30 (22), 1705779 10.1002/adma.201705779. 29659088

[ref30] Agrawal A.; Singh A.; Yazdi S.; Singh A.; Ong G. K.; Bustillo K.; Johns R. W.; Ringe E.; Milliron D. J. Resonant Coupling between Molecular Vibrations and Localized Surface Plasmon Resonance of Faceted Metal Oxide Nanocrystals. Nano Lett. 2017, 17 (4), 2611–2620. 10.1021/acs.nanolett.7b00404. 28337921

[ref31] Fano U. Effects of Configuration Interaction on Intensities and Phase Shifts. Phys. Rev. 1961, 124 (6), 1866–1878. 10.1103/PhysRev.124.1866.

[ref32] Yang W.; Liu Y.; Edvinsson T.; Castner A.; Wang S.; He S.; Ott S.; Hammarström L.; Lian T. Photoinduced Fano Resonances between Quantum Confined Nanocrystals and Adsorbed Molecular Catalysts. Nano Lett. 2021, 21 (13), 5813–5818. 10.1021/acs.nanolett.1c01739. 34132552

[ref33] Yang W.; Vansuch G. E.; Liu Y.; Jin T.; Liu Q.; Ge A.; Sanchez M. L. K.; Haja D. K.; Adams M. W. W.; Dyer R. B.; Lian T. Surface-Ligand “Liquid” to “Crystalline” Phase Transition Modulates the Solar H2 Production Quantum Efficiency of CdS Nanorod/Mediator/Hydrogenase Assemblies. ACS Appl. Mater. Interfaces 2020, 12 (31), 35614–35625. 10.1021/acsami.0c07820. 32662974

[ref34] Kirkwood N.; Monchen J. O. V.; Crisp R. W.; Grimaldi G.; Bergstein H. A. C.; du Fossé I.; van der Stam W.; Infante I.; Houtepen A. J. Finding and Fixing Traps in II–VI and III–V Colloidal Quantum Dots: The Importance of Z-Type Ligand Passivation. J. Am. Chem. Soc. 2018, 140 (46), 15712–15723. 10.1021/jacs.8b07783. 30375226 PMC6257620

[ref35] Kennehan E. R.; Munson K. T.; Grieco C.; Doucette G. S.; Marshall A. R.; Beard M. C.; Asbury J. B. Influence of Ligand Structure on Excited State Surface Chemistry of Lead Sulfide Quantum Dots. J. Am. Chem. Soc. 2021, 143 (34), 13824–13834. 10.1021/jacs.1c06248. 34420309

[ref36] Liu N.; Weiss T.; Mesch M.; Langguth L.; Eigenthaler U.; Hirscher M.; Sönnichsen C.; Giessen H. Planar Metamaterial Analogue of Electromagnetically Induced Transparency for Plasmonic Sensing. Nano Lett. 2010, 10 (4), 1103–1107. 10.1021/nl902621d. 20017551

[ref37] Herlihy D. M.; Waegele M. M.; Chen X.; Pemmaraju C. D.; Prendergast D.; Cuk T. Detecting the oxyl radical of photocatalytic water oxidation at an n-SrTiO3/aqueous interface through its subsurface vibration. Nat. Chem. 2016, 8 (6), 549–555. 10.1038/nchem.2497. 27219698

[ref38] Hanifi D. A.; Bronstein N. D.; Koscher B. A.; Nett Z.; Swabeck J. K.; Takano K.; Schwartzberg A. M.; Maserati L.; Vandewal K.; van de Burgt Y.; Salleo A.; Alivisatos A. P. Redefining near-unity luminescence in quantum dots with photothermal threshold quantum yield. Science 2019, 363 (6432), 1199–1202. 10.1126/science.aat3803. 30872520

[ref39] Takeda H.; Koike K.; Morimoto T.; Inumaru H.; Ishitani O., Photochemistry and photocatalysis of rhenium(I) diimine complexes. In Advances in Inorganic Chemistry; van Eldik R.; Stochel G., Eds.; Academic Press, 2011; Vol. 63, pp 137–186.

[ref40] Kiefer L. M.; Kubarych K. J. Solvent-Dependent Dynamics of a Series of Rhenium Photoactivated Catalysts Measured with Ultrafast 2DIR. J. Phys. Chem. A 2015, 119 (6), 959–965. 10.1021/jp511686p. 25607849

[ref41] Huang J.; Stockwell D.; Huang Z.; Mohler D. L.; Lian T. Photoinduced Ultrafast Electron Transfer from CdSe Quantum Dots to Re-bipyridyl Complexes. J. Am. Chem. Soc. 2008, 130 (17), 5632–5633. 10.1021/ja8003683. 18393497

[ref42] Zhu H.; Yang Y.; Wu K.; Lian T. Charge Transfer Dynamics from Photoexcited Semiconductor Quantum Dots. Annu. Rev. Phys. Chem. 2016, 67 (1), 259–281. 10.1146/annurev-physchem-040215-112128. 27215815

[ref43] Takeda H.; Koike K.; Inoue H.; Ishitani O. Development of an Efficient Photocatalytic System for CO2 Reduction Using Rhenium(I) Complexes Based on Mechanistic Studies. J. Am. Chem. Soc. 2008, 130 (6), 2023–2031. 10.1021/ja077752e. 18205359

[ref44] Gebre S. T.; Kiefer L. M.; Guo F.; Yang K. R.; Miller C.; Liu Y.; Kubiak C. P.; Batista V. S.; Lian T. Amine Hole Scavengers Facilitate Both Electron and Hole Transfer in a Nanocrystal/Molecular Hybrid Photocatalyst. J. Am. Chem. Soc. 2023, 145 (5), 3238–3247. 10.1021/jacs.2c13464. 36706437 PMC9912264

[ref45] Huang J.; Gatty M. G.; Xu B.; Pati P. B.; Etman A. S.; Tian L.; Sun J.; Hammarström L.; Tian H. Covalently linking CuInS2 quantum dots with a Re catalyst by click reaction for photocatalytic CO_2_ reduction. Dalton Transactions 2018, 47 (31), 10775–10783. 10.1039/C8DT01631C. 30019727

[ref46] Zhu H.; Song N.; Lian T. Wave Function Engineering for Ultrafast Charge Separation and Slow Charge Recombination in Type II Core/Shell Quantum Dots. J. Am. Chem. Soc. 2011, 133 (22), 8762–8771. 10.1021/ja202752s. 21534569

[ref47] Zhu H.; Song N.; Rodríguez-Córdoba W.; Lian T. Wave Function Engineering for Efficient Extraction of up to Nineteen Electrons from One CdSe/CdS Quasi-Type II Quantum Dot. J. Am. Chem. Soc. 2012, 134 (9), 4250–4257. 10.1021/ja210312s. 22329340

[ref48] Klimov V. I. Nanocrystal Quantum Dots, 2nd ed.; CRC Press: Boca Raton, 2010.

[ref49] Martinez-Gomez L.; Gebre S. T.; Lian T.; Ribeiro R. F. Theory of vibronic adsorbate-surface Fano resonances. arXiv 2024, 10.48550/arXiv.2410.11793.

