# Supplementary material for: Fano Resonance in CO2 Reduction Catalyst Functionalized Quantum Dots
Source: J Am Chem Soc. 2025 Mar 21;147(13):10966–73. doi: 10.1021/jacs.4c14499 (PMC11969527; doi:10.1021/jacs.4c14499)
Supplement: Supplementary file 1 — ja4c14499_si_001.pdf [file ja4c14499_si_001.pdf]

Supporting Information

Fano Resonance in CO_2 Reduction Catalyst Functionalized Quantum Dots

Sara T. Gebre†, Luis Martinez-Gomez†, Christopher R. Miller‡, Clifford P. Kubiak‡*, Raphael F. Ribeiro†*, Tianquan Lian†*

† Department of Chemistry, Emory University, Atlanta, Georgia 30322, United States

‡ Department of Chemistry and Biochemistry, University of California, San Diego, 9500 Gilman Drive, MC 0358, La Jolla, California 92093, United States

Contents

S1. Experimental Methods

S2. Characterization of ReC0A on CdSe QDs

S3. TA spectra of ReC0A on QDs

S4. Subtraction of solvent from TRIR spectra

S5. Fitted TRIR spectra of CdSe with and without ReC0A

S6. References

S1. Experimental Methods

Chemicals. Solvents and reagents used include oleic acid (OA), octadecene (ODE), cadmium oxide, tris(trimethylsilyl)phosphine, trioctylphosphine (TOP), trioctylphosphine oxide (TOPO), toluene, ethanol, acetonitrile, chloroform, hexanes, and heptane were all obtained from Sigma Aldrich. Octadecylphosphonic acid (ODPA) was acquired from PCI synthesis. Quantum dots were synthesized as described below, and ReC0A was synthesized according to previous procedures[1] by the Kubiak group at the University of California, San Diego.

CdSe Quantum Dot Synthesis. Several varying sizes of CdSe QDs were synthesized for this study according to previous procedure described by Hanifi *et al.* with some changes.[2] The two largest CdSe QDs were synthesized by mixing trioctylphosphine oxide (TOPO, 3 g), octadecylphosphonic acid (ODPA, 400 mg) and CdO (60 mg) in a three necked round bottomed flask. While stirring, the mixture was heated to 150 ^oC and left under vacuum for 30 min. After switching to argon, the heat was increased to 350 ^oC to dissolve the CdO. Once the solution became transparent, trioctylphosphine (TOP) was added (1.5 g) and the temperature raised to 370 ^oC. Meanwhile, a selenide solution was made in TOP (58 mg Se into 360 mg TOP) and sonicated. Once the mixture reached the desired temperature, the selenide solution was added to the reaction mixture. The reaction was allowed to proceed for varying amounts of time while observing color changes depending on the desired size. The larger sizes (580-620 nm) were obtained by waiting for the solution to turn an extremely dark brown color. The second largest size (545-550 nm) was obtained by waiting for the solution to turn a very dark red color. Once the corresponding color was observed, 3mL ODE was injected to stop the reaction and the round bottomed flask was cooled with a stream of air, then a water bath. The smallest size of CdSe was synthesized with this procedure except the amount of ODPA added was 280 mg and the reaction was stopped once the solution turned orange. For each QD, once the reaction stopped, the color of the solution reverted to a lighter version. In order of size (smallest to largest), the QDs were yellow, bright red, and dark red. Once cooled, a small amount of toluene was added to the flask, after which the QD solution was split between two 15 mL falcon tubes. An excess amount of ethanol was added to each tube to aid in the precipitation of the QDs. The solution in both falcon tubes was centrifuged for 5 min at 5500 rpm. Once finished, the supernatant was disposed of and the precipitated QDs were redispersed in ~ 2 mL of toluene with excess ethanol added afterwards. This centrifugation

procedure was performed three times to obtain washed QDs. After the last centrifugation and removal of supernatant, the pellet was dissolved in hexanes and centrifuged one last time. The resultant supernatant was comprised of QDs and were removed and transferred to a vial, avoiding any pellets that had formed. The resultant CdSe QDs had exciton absorptions at 490 nm (yellow), 545/550 nm (bright red), and 580/582/620 nm (dark red to dark brownish) corresponding to diameters ranging from 2.3 nm to 5.6 nm.

Sample Preparation. Samples for both transient absorption and time resolved IR were prepared the same way, with a difference of solvents. The day before experiments were performed, ReC0A in MeCN was rotary evaporated depending on the desired concentration. The QD in hexanes was then added to the same vial on top of the dried ReC0A and rotary evaporated once again. The dried samples were stored overnight. On the experiment day, the dried sample was redissolved in 1 mL of either heptane (TA) or hexanes (TRIR) and sonicated for 2 hours. The amount of QD rotary evaporated corresponded to an OD of 0.3 determined from UV-Vis. Four concentrations of ReC0A were added to each QD: 0.25x, 0.5x, 1x, and 2x. The volumes of ReC0A used corresponded to the desired amount of catalyst adsorbed to the surface. For example, if 1xRe was desired, 1 mL of ReC0A in MeCN (0.18 OD at 400 nm) was used to obtain a ~ 1:2 ratio of ReC0A to QD according to a 0.3 OD absorbance in the UV-Vis. For TA experiments, the prepared samples were transferred to Starna 1 mm optical glass cuvettes and stirred for experiments. For TRIR, samples were injected into a Harrick FTIR cell with a spacer of 950 um and stirred as well. Both UV-Vis (Agilent 8453) and FTIR (ThermoFisher Nicolet is50) were taken before each TRIR experiment, and UV-Vis before TA.

Visible Femtosecond Transient Absorption Spectroscopy. The femtosecond transient absorption measurements were conducted in a Helios spectrometer (Ultrafast Systems LLC) with pump and probe beams derived from a regenerative amplified Ti: Sapphire laser system (Coherent Astrella, 35 fs, 4 mJ/pulse, and 1 kHz repetition rate). The 800 nm output pulse was split into two beams with a beam splitter. One beam passed through a tunable optical parametric amplifier (OperA solo, Coherent) to generate a tunable visible pump. During the measurement, the pump beam was chopped by a synchronized chopper to 500 Hz. The other beam was attenuated and focused on either a sapphire or CaF_2 window to generate the white light continuum with a wavelength range from 350 nm to 800 nm, referred to as probe beam. The probe beam was focused into a 1-mm path

length quartz cuvette (Starna) containing the sample in heptane. The transmission of the probe was collected by a fiber optics-coupled multichannel spectrometer with complementary metal-oxide-semiconductor (CMOS) sensors and detected at a frequency of 1 kHz (Ultrafast systems, Helios). The delay between the pump and probe pulses was controlled by a motorized delay stage. Samples in 1-mm cuvettes were used for all spectroscopy measurements and stirred vigorously during the measurements. The data were analyzed with Surface Xplorer.

Transient Infrared Absorption Spectroscopy. TRIR experiments were conducted using a commercial Ti:Sapphire regenerative amplifier (Astrella, Coherent) at 800 nm with a repetition rate of 1 kHz and pulse duration of ~35 fs. An optical parametric amplifier (OperA solo, Coherent) pumped by the regenerative amplifier was used to provide a pump beam with tunable wavelengths. Another optical parametric amplifier (OperA solo, Coherent) pumped by the regenerative amplifier was used to generate a tunable IR probe via difference frequency generation (DFG) by signal and idler beams. The pump beam was chopped by a synchronized chopper to 500 Hz and focused on a 950-um path length Harrick cell containing the sample in hexanes. The IR probe, which was also focused at the sample, overlapped with the temporally delayed pump beam controlled by a movable delay stage. The probe intensity with and without pump was analyzed by a Teledyne/Phasetech nitrogen-cooled 128×128 MCT detector to generate the ΔA spectrum.

S2. Characterization of ReC0A on CdSe QDs

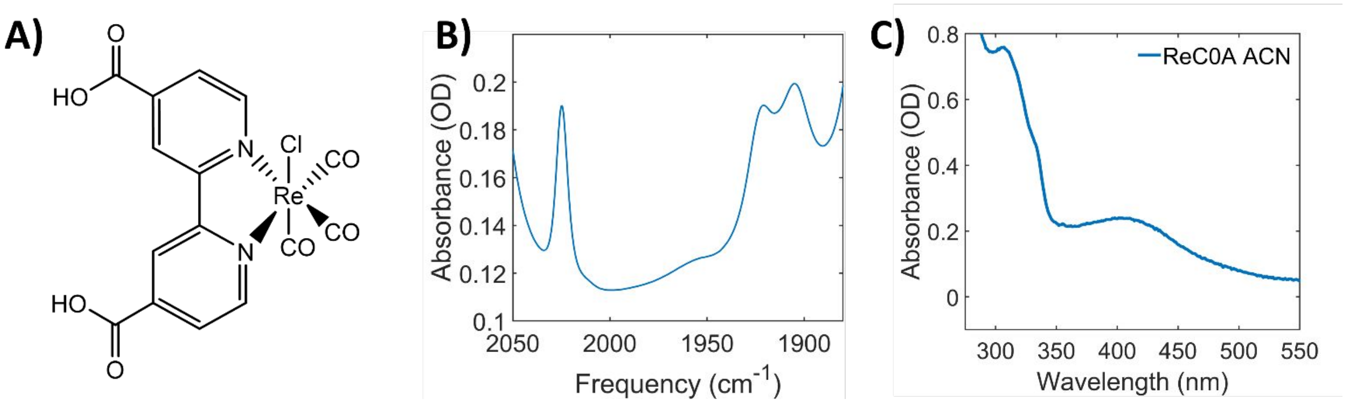

Figure S1. A) Molecular structure of ReC0A. Carboxylic acid groups allow binding of the catalyst to the QD surface. B) FTIR of ReC0A in acetonitrile shows high and low frequency modes corresponding to different CO stretching modes (symmetric, asymmetric, and out of phase

symmetric, respectively). C) UV-Vis spectra of ReC0A in MeCN. Absorptions at approximately 305 and 400 nm correspond to the π to π^* transition of the bipyridine and the MLCT transition, respectively.

Table S1. Number of ReC0A molecules bound to the QD surface.

	**0.25xRe**	**0.5xRe**	**1xRe**	**2xRe**
**CdSe490**	1.2	3.3	5.9	8.4
**CdSe545**	--	10.1	17.8	36.7
**CdSe582**	9.4	9.7	14.7	25.6

In order to obtain the number of ReC0A on the QDs, the catalyst absorbances in the FTIR were used to calculate the concentrations of ReC0A for each QD. After obtaining the QD concentrations, the number of moles for each were converted to the number of molecules or QDs in each sample. The number of ReC0A molecules was divided by the number of QDs in the sample volume to get the average amount of catalyst on each QD. For CdSe545, an additional concentration, 4xRe, was also tested, with FTIR shown in Figure S2a (green trace). This concentration had approximately 65.5 molecules on the QD surface. CdSe545-0.25xRe is not included because this sample was not tested due to a sample preparation error where the catalyst concentrations were doubled. Instead, we have included a higher concentration, CdSe545-4xRe.

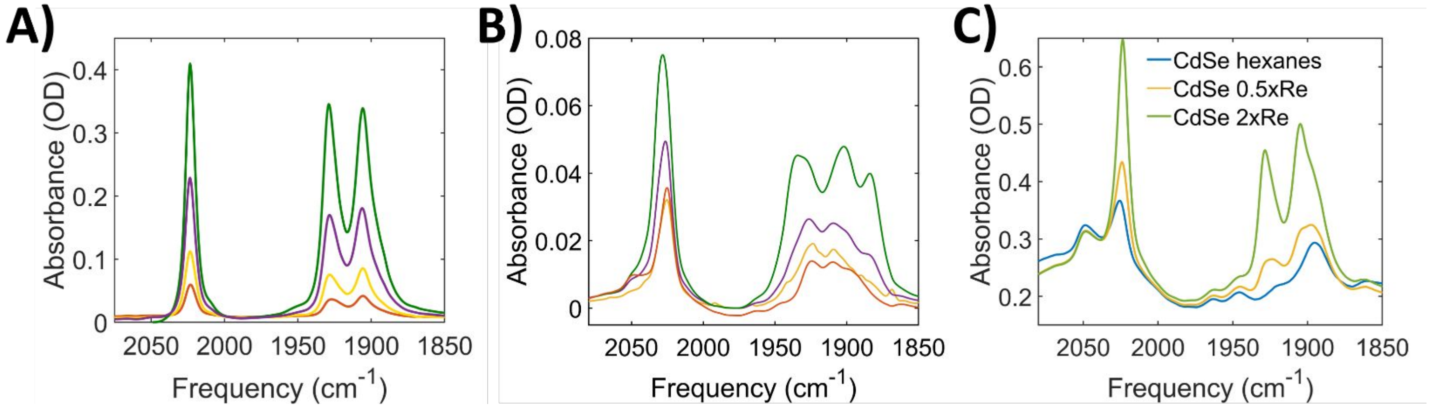

Figure S2. FTIR of varying concentrations of ReC0A on the QD. A) Varying ReC0A concentrations on CdSe545 where higher concentrations were used: 0.5x (red), 1x (yellow), 2x (purple), and 4xRe (green). B) Varying ReC0A concentrations on CdSe582 (red: 0.25x, yellow: 0.5x, purple: 1x, green: 2x). C) FTIR of CdSe545 0.5x and 2xRe compared to the QD in hexanes demonstrates the overlap of solvent and ReC0A IR absorption bands.

S3. TA spectra of ReC0A on QDs

The following figures show TA spectra of the QDs with each concentration of ReC0A bound. Each QD spectra demonstrates slow bleach recovery indicating a long-lived band edge electron within 1.6 ns. Lifetimes in Table S2 and kinetics traces in Figure 2 demonstrate that the exciton lives on the order of nanoseconds. CdSe490-ReC0A shows fast bleach recovery within 1.6 ns indicating fast electron transfer from the QD to the catalyst. However, no signal corresponding to the singly reduced catalyst appears (~520 nm) due to the strong absorption of the QD bleach. CdSe545 spectra with ReC0A demonstrate the same phenomenon as seen with CdSe490. However, because of the increased size, the electron transfer does not proceed as quickly, resulting in slower bleach recovery compared to CdSe490. The last QD, CdSe582, shows that with the catalyst, there is little bleach recovery, meaning that there is very little electron transfer occurring.

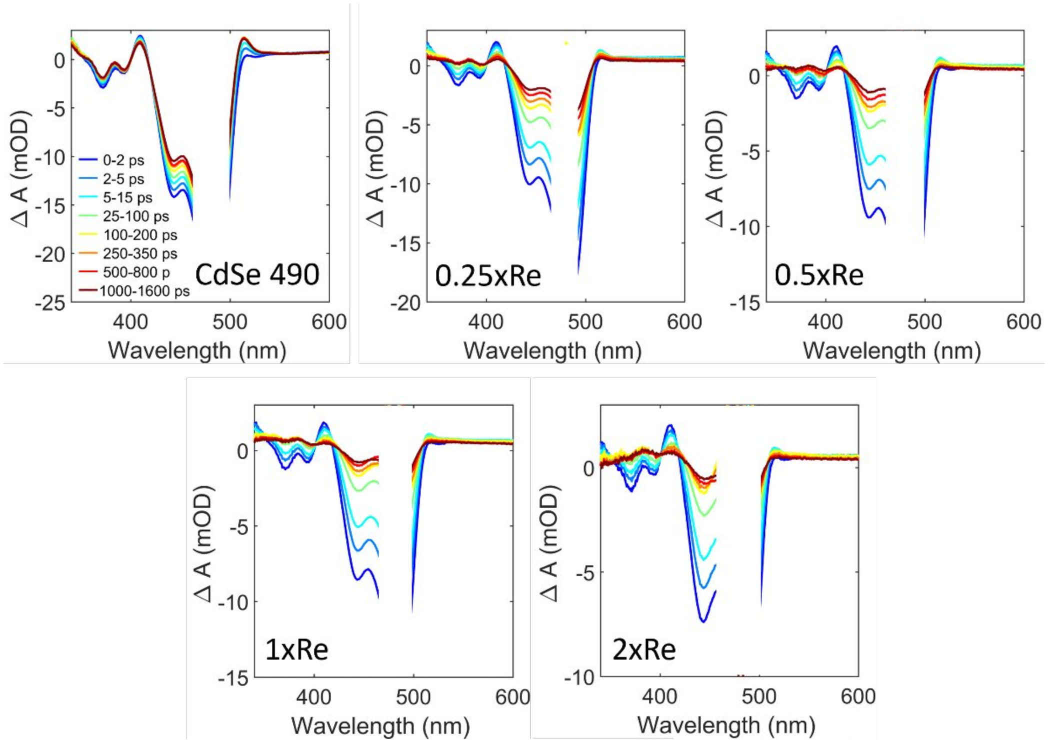

Figure S3. TA spectra for CdSe490 with varying amounts of ReC0A.

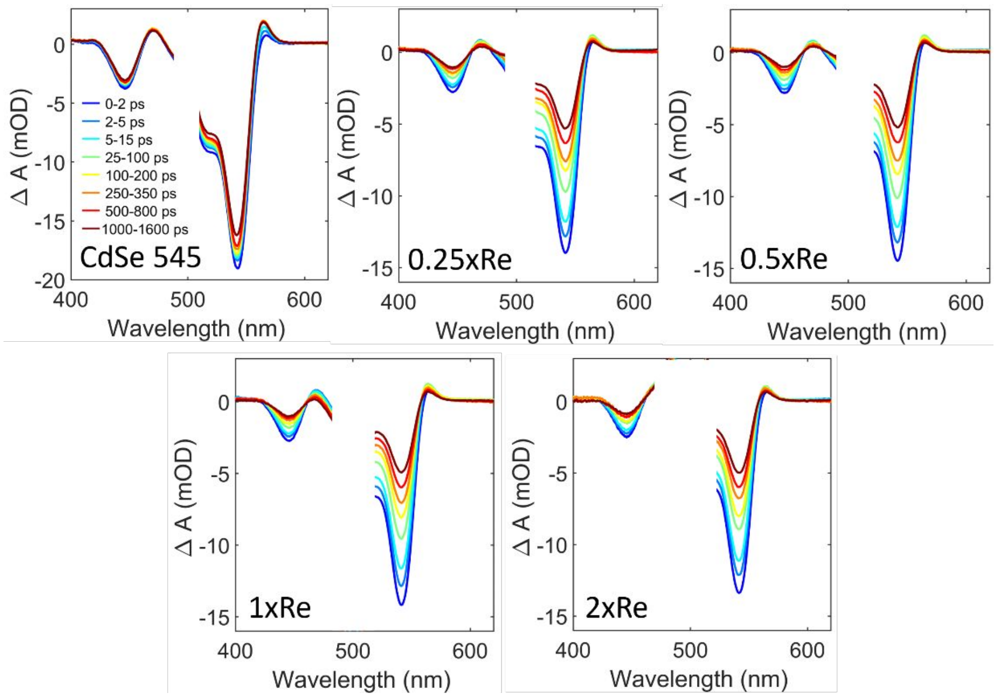

Figure S4. TA spectra for CdSe545 with and without ReC0A bound.

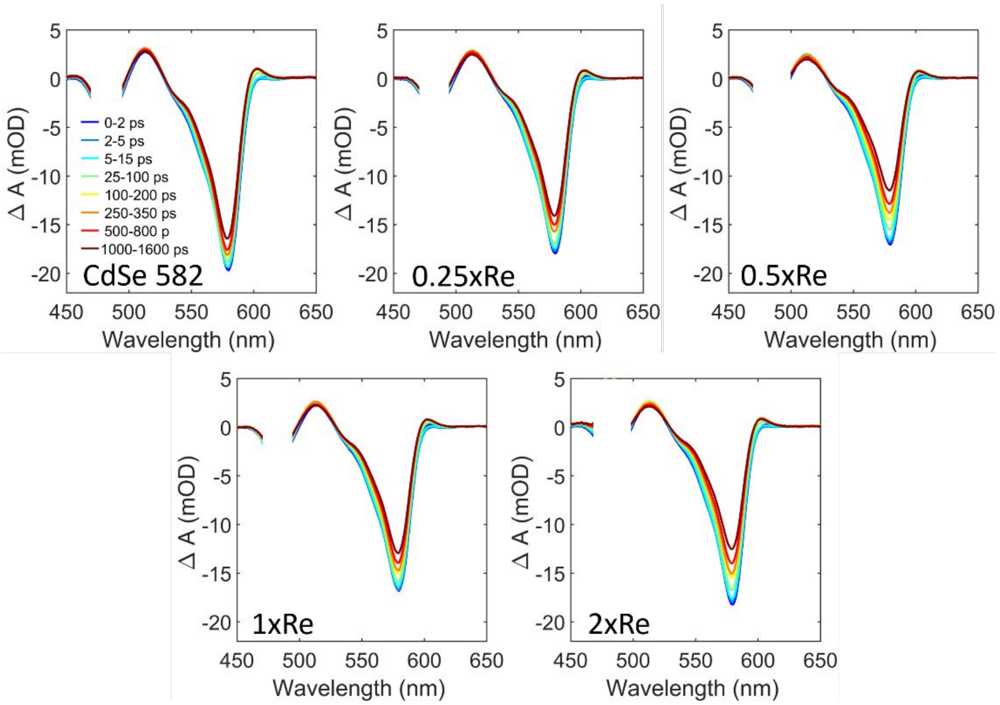

Figure S5. TA spectra for CdSe582 with and without ReC0A.

Table S2. Amplitude weighted average time constants of CdSe QDs with and without ReC0A

	**CdSe490**	**CdSe545**	**CdSe582**
QD only	13.8 ± 0.454 ns	13.1 ± 1.62 ns	7.38 ± 0.266 ns
0.25xRe	0.685 ± 0.058 ns	1.44 ± 0.057 ns	6.53 ± 1.06 ns
0.5xRe	0.141 ± 0.018 ns	1.17 ± 0.043 ns	4.63 ± 0.878 ns
1xRe	0.103 ± 0.019 ns	0.994 ± 0.038 ns	6.24 ± 1.33 ns
2xRe	0.137 ± 0.033 ns	1.40 ± 0.104 ns	4.40 ± 0.326 ns

S4. Subtraction of solvent from TRIR spectra

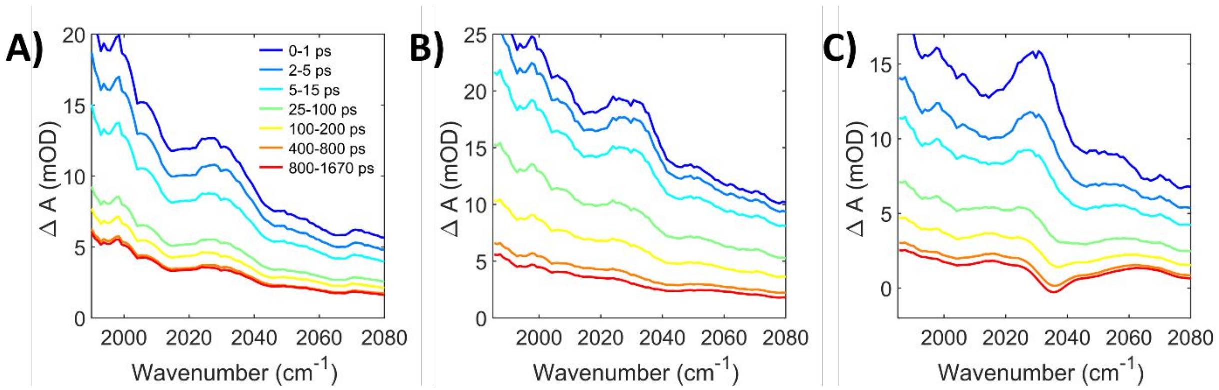

Figure S6. Unsubtracted TRIR spectra. A) CdSe490 in hexanes. The two large absorptions observed correspond to solvent FR coupling. B) CdSe490-0.25xRe. Amplitude of FR signal at ~2030 cm^{-1} increased compared to A, suggesting FR coupling with the ReC0A. The solvent and ReC0A signals overlap. C) CdSe490-2xRe. The amplitude of FR is even larger, due to increased ReC0A concentration. Solvent FR signal can be seen at 2050 cm^{-1}. This will allow us to subtract the solvent contribution from the 2030 cm^{-1} peak.

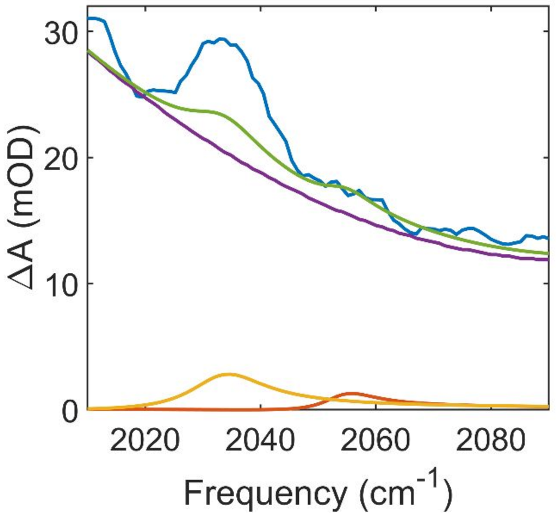

**Parameters**	**Values from solvent fit**
Amplitude 2	0.00012
q_2	3.13 (**fixed**)
$v_{0,2}$	2054 (**fixed**)
Γ_2	11 (**fixed**)
Amplitude 1 (A_2/1.42)	0.000085
q_1	5.69 (**fixed**)
$v_{0,1}$	2033 (**fixed**)
Γ_1	16 (**fixed**)

Figure S7. Demonstration of how solvent contribution is subtracted from TRIR spectra. Blue: unsubtracted data; Purple: QD background fitted with a polynomial (n=3); Red: fit for second FR

peak; Yellow: solvent peak to subtract from QD-ReC0A, determined by the amplitude ratio between A_{2050} and A_{2030} from the solvent fit (Ratio = 1.42); Green: FR signal originating from the solvent. This will be subtracted from the data.

Since hexanes appears to couple to the QDs, we globally fit the data to equation 1 with two Fano and two Lorentzian terms corresponding to each peak, 2030 and 2050 cm^{-1}. After ReC0A is bound to the QD, because the solvent and ReC0A CO symmetric mode overlap, each QD-hexanes sample was fit to equation 1 with two Fano terms and two Lorentzian terms to obtain q values for coupling of both solvent vibrations. In order to separate these contributions, we subtracted the solvent peaks from each QD-ReC0A sample spectra. We first fit the QD-hexanes spectra and obtained q, as well as the amplitudes of the FR peaks. The Lorentzian amplitudes were held to some small value (~0.001) due to a negligible contribution of the Lorentzian absorption (from FTIR absorbance) in comparison to the FR signal. To subtract the solvent contribution, we held the parameters from the solvent fit constant (q_i, ε_i, $\nu_{0,i}$, and Γ_i) and found the ratios between the fitted amplitude of the 2050 cm^{-1} peak to the 2030 cm^{-1} absorption (A_{2050}/A_{2030}). The ratio was fixed for each time delay when inputting amplitude values for the QD-ReC0A FR peak and the hexanes peak at 2050 cm^{-1}. For example, if the ratio between the peaks was $A_{2050}/A_{2030} = 2.0$ from the CdSe490-hexanes sample at the 0-1 ps time delay, after moving to the QD-1xRe sample, all other fitting parameters would be held constant. If the Fano amplitude for 2050 cm^{-1} (hexanes) was 0.02, then the amplitude of the hexanes under the QD-1xRe FR peak would be 0.04, after which we would be able to subtract this curve from the spectra to result in the QD-ReC0A only FR peak (Figure S7). All other spectra that appear after Figure S7 have been solvent subtracted. Within the 2015 to 2060 cm^{-1} region, there may be artifacts of the solvent subtraction. Other similar features may be seen at frequencies below 2000 cm^{-1}, which we attribute to noise due to the lack of infrared light reaching the detector in that region.

In addition, errors associated with the q values were obtained from the fit. These uncertainties are the estimated standard deviation of the fit coefficients originating from the residuals (of the data compared to the fit).[3] More specifically, Igor, the fitting software utilized, uses a maximum likelihood estimation where the probability of the parameters in our fit is

maximized assuming the measurement errors are independent and normally distributed and minimizes the chi squared. Further details can be found in Ref [3].

S5. Fitted TRIR spectra of CdSe with and without ReC0A

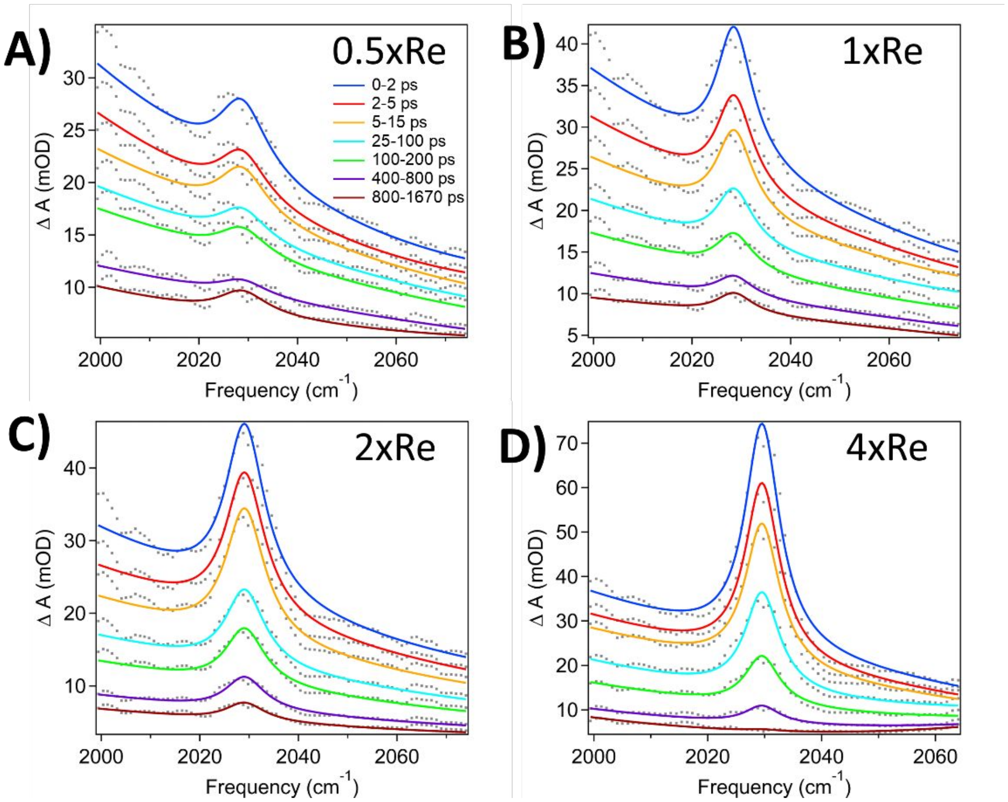

Figure S8. CdSe545 with ReC0A. As seen with CdSe490, with higher amounts of ReC0A added to the QDs, the FR signal increases. 4xRe is the highest concentration used in this study and shows a very clear sharp FR signal. CdSe545-0.25xRe was not included in this study.

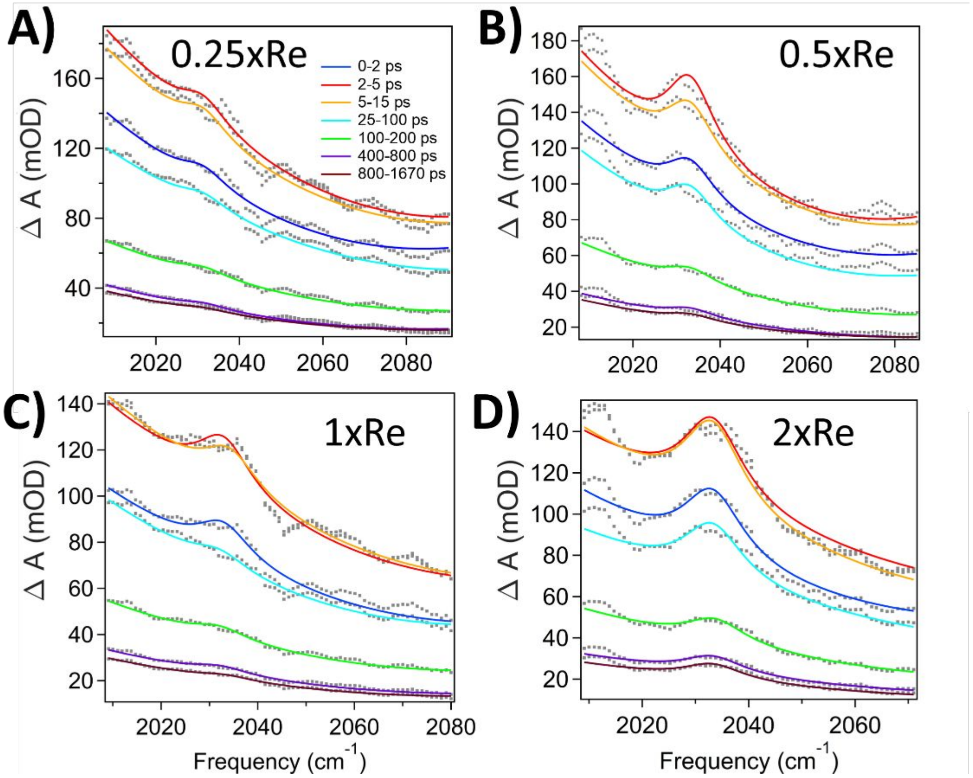

Figure S9. CdSe582 ReC0A. The same trend that was seen for the other two QDs is observed for CdSe582.

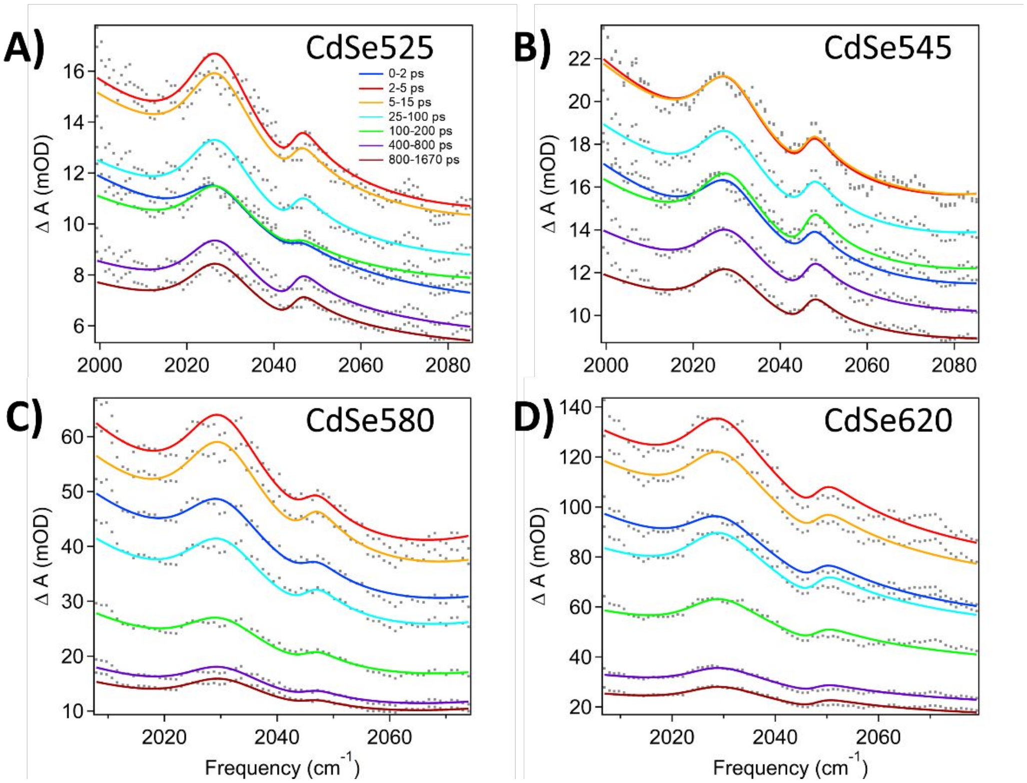

Figure S10. TRIR of QDs in hexanes. Both solvent absorptions are seen for each QD for our size dependent study.

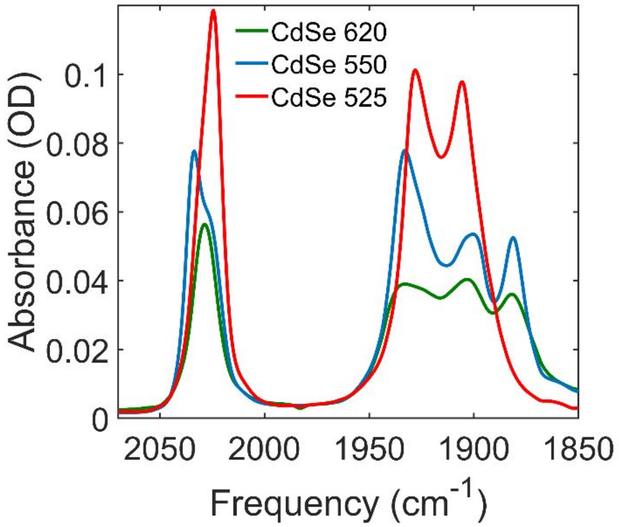

Figure S11. FTIR of CdSe525, CdSe550, and CdSe620 with 2xRe bound to the surface.

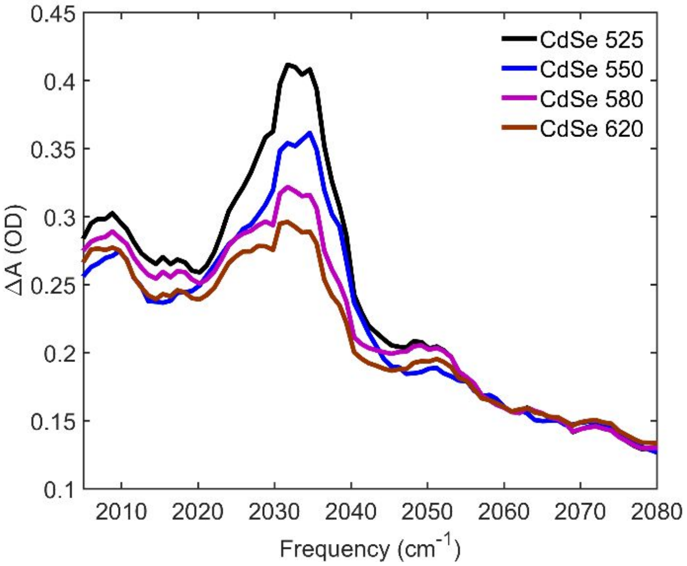

Figure S12. Transient infrared spectra (0-1 ps) of each CdSe QD (525, 545, 580, 620) with 2xRe bound to the surface. Each trace has been scaled to the QD electron signal so that the trend in FR amplitude as a function of QD size may be seen clearly. The FR amplitude increases with decreasing QD size.

Table S3. Number of ReC0A molecules bound to CdSe525, CdSe550, CdSe580, and CdSe620.

	**CdSe525**	**CdSe550**	**CdSe580**	**CdSe620**
**2xRe**	21.8	17.3	25.6	27.7

Table S4. Solvent *q* values for all QDs in hexanes.

**CdSe**	**490**	**525**	**545**	**550**	**580**	**582**	**620**
**2030 cm^{-1}**	3.7 ± 0.08	6.7 ± 0.41	6.7 ± 0.33	6.5 ± 0.4	5.6 ± 0.21	5.7 ± 0.26	4.3 ± 0.18
**2050 cm^{-1}**	0.94 ± 0.05	1.6 ± 0.1	3.0 ± 0.31	1.9 ± 0.1	2.0 ± 0.13	3.1 ± 0.15	1.0 ± 0.06

S6. References

1. Komreddy, V.; Ensz, K.; Nguyen, H.; Paul Rillema, D., Synthesis and characterization of rhenium(I) 4,4′-dicarboxy-2,2′-bipyridine tricarbonyl complexes for solar energy conversion. *Inorganica Chimica Acta* **2020,** *511*, 119815.
2. Hanifi, D. A.; Bronstein, N. D.; Koscher, B. A.; Nett, Z.; Swabeck, J. K.; Takano, K.; Schwartzberg, A. M.; Maserati, L.; Vandewal, K.; van de Burgt, Y.; Salleo, A.; Alivisatos, A. P., Redefining near-unity luminescence in quantum dots with photothermal threshold quantum yield. *Science* **2019,** *363* (6432), 1199-1202.
3. Press, W. H. T., Saul A.; Vetterling, William T.; Flannery, Brian P.;, Numerical Recipes in C: The Art of Scientific Computing. In *Chapter 15: Modeling of Data* [Online] Second ed.; Cambridge University Press: Cambridge, 1992; p. 1018.
